# High prevalence of helminths infection and associated risk factors among adults living in a rural setting, central Kenya: a cross-sectional study

**DOI:** 10.1186/s41182-017-0055-8

**Published:** 2017-07-01

**Authors:** Janet Masaku, Faith Mutungi, Paul M. Gichuki, Collins Okoyo, Doris W. Njomo, Sammy M. Njenga

**Affiliations:** Eastern and Southern Africa Centre of International Parasite Control (ESACIPAC), Kenya Medical Research Institute (KEMRI), P.O. Box 54840, Nairobi, 00200 Kenya

**Keywords:** Schistosomiasis, *S. mansoni*, Prevalence, Risk factors, Soil-transmitted helminths in Kenya

## Abstract

**Background:**

Schistosome infection and soil-transmitted helminths (STHs) are major public health problems in many developing countries where they contribute to the suffering of populations living in poor settings. A cross-sectional survey was conducted in four rural villages in central region of Kenya to provide information on the status of schistosome and STH infections. Previous studies conducted in the area among primary school children showed that there were high STH and *Schistosoma mansoni* infections. This paper presents the results of a parasitological investigation and the associated risk factors of infection among adults living in the study villages.

**Methods:**

A total of 495 adults (18–84 years) from systematically selected households were sampled during this cross-sectional survey. They were interviewed and screened for *S. mansoni* and STHs using duplicate Kato-Katz thick smears. Comparison of prevalence by age group and gender was explored by confidence interval plots, and 95% CI were obtained by generalized least squares (GLS) random effects model. Risk factors associated with *S. mansoni* infection were determined using mixed effects logistic regression at 95% CI taking into account household clusters.

**Results:**

The study revealed that the prevalence of *S. mansoni* infection was 33.5% (95% CI 29.6–38.0) among adults in the study villages, while the prevalence of STH infection was 0.2% (95% CI 0–1.4) with hookworm being the only detected STH species. However, the village and education level were the only risk factors which showed significant evidence of association with *S. mansoni* infections.

**Conclusions:**

The current study shows that adult communities in the study area were highly infected with *S. mansoni*. The study suggests that it may be necessary to develop contemporary approaches towards preventive chemotherapy interventions to adults in high endemic areas to complement the ongoing school-based deworming programme.

**Electronic supplementary material:**

The online version of this article (doi:10.1186/s41182-017-0055-8) contains supplementary material, which is available to authorized users.

## Background

Schistosome and soil-transmitted helminth (STH) infections are among the neglected tropical diseases (NTDs) that cause a huge burden of disease in the developing world [1]. Indeed, more than half of the human population is at risk of schistosomiasis and STHs with more than 1 billion people infected, possibly causing a global burden of more than 40 million disability-adjusted life years (DALYs) lost annually [2–7]. Improvements have been made to reduce helminth transmission in many parts of the world [8], but worm infections continue to be an issue of major public health and socio-economic concern. Helminthic diseases disproportionately affect those living in poverty [9, 10], with the poorest of the poor commonly suffering from multiple species infections concurrently [11–13]. Among the health effects associated with these parasites are growth retardation, intestinal obstruction, hepatic and biliary diseases, impaired cognitive development and nutritional difficulties, such as iron deficiency and anemia [14, 15].

In Kenya, over 6 million people are estimated to be infected with schistosomes [1] and many more are at risk of infection. The highest schistosome infection rates are found in those aged 10–19 years, but adult workers in rural areas who either work or are employed in activities associated with water contact are also affected [16–18]. Overall, the prevalence of schistosome infection ranges from 5 to over 65% in communities in Kenya and contributes to significant morbidity [19–21]. In most cases, schistosome infections co-occur with STHs. The prevalence of STHs in Kenya is predominantly attributed to hookworm, *Ascaris lumbricoides* and *Trichuris trichiura* [22]. It is estimated that approximately 10 million Kenyans are infected with STHs and over 12 million people living in rural endemic areas in the country are at risk of infection with these parasites [23]. In the central region of Kenya, schistosomiasis is primarily caused by *S. mansoni* and it is mainly associated with the Mwea irrigation scheme where rice farming is done. Previous studies conducted in the area showed that there was a high prevalence of *S. mansoni* infection (above 50%) among primary school children 2 years after withdrawal of a 5-year mass drug administration (MDA) programme [24].

Currently, school-based deworming is the primary approach being used for mass treatment of schistosome and STH infections and has been supported in a few areas in the country, mostly by local and international non-governmental organizations (NGOs). However, this treatment is only limited to pre-school children and school-age children. Effective control by the school-based strategy may not be possible if other infected members of the community are not treated, such as farmers in irrigation schemes, sand harvesters and mothers of childbearing age who can have high levels of infection but are not reached by school-based treatment programmes [25].

Despite the health importance of schistosome and STH infections in Kenya, a national control programme for the community has not been instituted. Nevertheless, a national deworming programme targeting school-aged children was launched by both Ministries of Education and Health and implemented in 45 districts (total districts 254) in the year 2009 [26]. To ensure a wider coverage of treatment, it is important to include the wider community who are also at risk of helminth infection in the control programmes. The guidelines developed by the World Health Organization (WHO) to control helminth infection in the community suggest three key approaches which include improved sanitation, health education and mass chemotherapy.

Effective and safe drugs for treatment of STHs are available in Kenya [25]. However, drugs for treatment of schistosome infection are not available in the health facilities in the country, and STH treatment is intermittently available in health facilities, and in other cases, it is distributed to the community members by community health extension workers (CHWEs). Most individuals at high risk of morbidity do not have access to treatment because of the poor health infrastructure, lack of awareness about the disease or cost. Previous studies conducted in Central Kenya achieved recommendable results in the prevention and control of these infections among school-age children through MDAs and health education [27]. However, involvement of communities, for both implementation of school-based treatment and for increased coverage to all those at risk, has not been effectively employed in this area with no published report on the level of infection. Therefore, the current study was initiated with the objective of determining the prevalence and intensity of *S. mansoni* and STH infections and the associated risk factors among adults living in a rural setting of Kirinyaga county, Central Kenya.

## Methods

### Study area and population

The study was conducted in Mwea irrigation scheme located in Mwea West Sub-county, Kirinyaga County, Central Kenya. Administratively, Kirinyaga County has five sub-counties, namely Kirinyaga East, Kirinyaga West, Mwea East, Mwea West and Kirinyaga Central. The county is located about 100 km north east of Nairobi, Kenya. It covers an area of 1478.1 km^2^ with an estimated 154,220 households and a total population of 528,054 persons [28]. The mean annual rainfall in this area is in the range of 1200–1600 mm per year. Mwea West Sub-County, where the study was conducted, has two locations (Kangai and Thiba) with a total of seven villages. The main socio-economic activity in this area is rice farming, which is done by gravity flow irrigation using water from river Thiba and Nyamindi. Mwea West Sub-County is endemic for both *S. mansoni* and STH infections. The target population was 502 adults aged above 18 years in four villages which were purposively sampled. The selected villages were Kiratina, Mbui Njeru, Mianya A and Mianya B. Mianya A and Mianya B villages neighbour each other, but administratively, they are two different villages. The sub-county was purposefully sampled based on endemicity of STH and *S. mansoni* infections owing to previous studies conducted in the neighbouring schools in the area [27]. All the participating adults were interviewed and stool samples collected from them.

### Study design

This cross-sectional study was conducted from 11^th^ June to 12^th^ September 2014. The inclusion criteria of the villages included location within the irrigation scheme and neighbouring a school which participated in the MDA programme. Four villages were purposively sampled (Mianya A, Mianya B, Kiratina and Mbui Njeru) since previous studies conducted in the area had shown a high prevalence of *S. mansoni* and STH infections in the neighbouring schools [27]. The study adopted probability proportional to size sampling (PPS) to determine the number of households to be selected per village. A total number of 2280 adults (above 18 years) met the inclusion criteria and were targeted to participate in the study. The inclusion criteria to participate in the study were to be above 18 years old, able to give written informed consent and have been residing within the selected villages in the last 3 months. The sample size for the study was calculated to 502 using the formula by Fisher et al. [29]. Sampling was based on practical feasibility. Each study village was mapped, divided into sectors based on topography and closeness to the source of infection, houses numbered and each individual registered according to the household. Systematic sampling was used to select households within each village whereby, every fourth household in each of the four villages was included. The first household was randomly selected. Household heads or their representatives from the sampled households in each village were interviewed, with those who were not present during the interview and subsequent follow-up visits classified as permanently missing and excluded from the study. Prior to the survey, meetings were held in all the villages with the community members and the local administration to communicate the study purpose and to obtain their consent. A written informed consent for participation in the study was obtained from each study participant before conducting the study.

### Sample collection and examination

All adults who gave written informed consent were provided with poly pots (stool containers) a day before the survey and requested to give their own fresh stool sample on collection day. Two trained field workers guided the study participants on stool sample collection during container distribution. They also visited the household of the selected study participants in the morning with a registration sheet to collect stool samples. All samples were obtained between 7:00 a.m. and 10:00 a.m. After collection, stool samples were given unique identification numbers. The name, sex, age and household number and village of the study participant were recorded. Screening of STHs and schistosome’s ova was based on duplicate Kato-Katz thick smears of 41.7 mg prepared from fresh stool samples to determine the prevalence and intensity of *S. mansoni* and STH infections [30]. This procedure was carried out at Kimbimbi sub-county hospital by trained medical laboratory technologists from the Ministry of Health (MOH).

### Questionnaire survey/household information survey

A structured pre-tested questionnaire developed in English and translated into the local dialect (Kikuyu) language was administered to all the adults recruited for the study. The questionnaire was made up of socio-demographic indicators which included the name, age, sex, marital status, occupation and education level which was categorized by the following: no education (those who have never been to school), primary level, secondary level and tertiary/college level. Environmental indicators like type of housing (wall and floor) and source of water for drinking were also determined.

### Data management and analysis

The data collected was counter-checked for accuracy and verified before double entry into a computer Excel spreadsheet. All statistical analyses were carried out using STATA version 12.0 (STATA Corporation, College Station, TX, USA). The observed overall prevalence and intensity of *S. mansoni* and STH infections was calculated by village, gender and age group levels. The 95% confidence intervals (95% CI) were obtained by binomial logistic regression. Comparison of prevalence by age group and gender was explored by confidence interval plots, and 95% CI were obtained by generalized least squares (GLS) random effects model. Infection intensities were classified into light, moderate and heavy infections, according to WHO guidelines [31] (Additional file 1: Table S1), and the prevalence of light to heavy infection together with 95% CIs were obtained using binomial. Factors associated with *S. mansoni* infection were analyzed using univariable analysis and the strength of the association measured as odds ratio (OR) using mixed effects logistic regression at 95% CI. To select minimum adequate variables for multivariable analysis, an inclusion criterion of *p* < 0.3 was pre-specified. Adjusted OR (aOR) were obtained by mutually adjusting all minimum generated variables using multivariable mixed effects logistic regression at 95% CI, with values considered significant at *p* < 0.05. For the purposes of this analysis, the following age groups were used: 18–29, 30–41, 42–53, 54–65 and >65 years old.

## Results

Out of the 502 sampled participants, 495 household heads across the four selected villages in Kirinyaga county consented to the study and hence their data were included in the analysis. The mean age of the participants was 38 years (standard deviation ± 12 years) with an age range of 18–84 years, and 70.5% were female. In each household, there was an average of two occupants at the time of the visit, with a range of 1 to 5 persons.

Table 1 summarizes the demographic characteristics, prevalence and mean intensity of *S. mansoni* infection as well as the risk factors associated with *S. mansoni* infection. The overall prevalence of *S. mansoni* infection was 33.5% (95% CI 29.6–38.0) with a mean intensity of 131 epg (95% CI 89–194). Over half, 54.1% (95% CI 45.5–64.2), of the participants in Mianya B village were infected, while Mbui Njeru village had the least infection levels. Similarly, high levels of infections were seen among the participants who did not have any level of education, 58.8% (95% CI 44.2–78.3). Those who do farming as their main economic activity were 34.2% (95% CI 30.1–39.0), those who lived in wood-walled houses were 47.3% (95% CI: 39.4–56.8), and among those using rainwater as their main source of water were 66.7% (95% CI 35.9–85.0).

**Table 1:** Prevalence (%), mean intensity (epg) and risk factors associated with *S. mansoni* infection

Category	No. examined (*N* = 495) *N* (%)	Prevalence (%) (95% CI)	Mean intensity (epg) (95% CI)	Multivariable logistic	
				aOR (95% CI)	*p* value
Overall	495 (100)	33.5 (29.6–38.0)	131 (89–194)	–	
Village					
Kiratina	130 (26.3)	26.2 (19.6–35.0)	31 (20–48)	Reference	
Mbui Njeru	136 (27.5)	13.2 (8.6–20.4)	15 (8–25)	0.54 (0.27–1.12)	0.097
Mianya A	118 (23.8)	45.8 (37.6–55.7)	164 (110–244)	2.09 (1.15–3.77)	0.015
Mianya B	111 (22.4)	54.1 (45.5–64.2)	357 (197–646)	4.38 (2.02–9.48)	<0.001
Gender					
Male	146 (29.5)	28.1 (21.6–36.4)	65 (39–106)	Reference	
Female	349 (70.5)	35.8 (31.1–41.2)	159 (102–249)	1.48 (0.92–2.37)	0.109
Age group					
18–29	123 (24.9)	38.2 (30.5–47.9)	123 (79–192)	–	
30–41	198 (40.0)	30.3 (24.5–37.5)	65 (44–98)	–	
42–53	110 (22.2)	30.0 (22.5–40.0)	87 (48–159)	–	
54–65	52 (10.5)	42.3 (30.7–58.3)	511 (217–1201)	–	
>65	12 (2.4)	33.3 (14.5–76.9)	62 (21–183)	–	
Marital status					
Married	368 (74.3)	34.0 (29.5–39.2)	139 (88–218)	Reference	
Unmarried	127 (25.7)	32.3 (25.1–41.6)	110 (53–230)	0.83 (0.51–1.35)	0.452
Education level					
No education	34 (6.9)	58.8 (44.2–78.3)	508 (201–1288)	2.31 (1.04–5.11)	0.039
Primary	352 (71.1)	31.5 (27.0–36.8)	119 (75–188)	Reference	
Secondary	103 (20.8)	33.0 (25.0–43.5)	56 (36–88)	1.74 (1.02–2.96)	0.041
College and above	6 (1.2)	16.7 (2.3–18.3)	4 (1–28)	0.74 (0.08–7.21)	0.797
Occupation					
Unemployed	4 (0.8)	25.0 (3.5–77.5)	54 (8–383)	–	
Farming	444 (89.7)	34.2 (30.1–39.0)	137 (91–208)	–	
Business	42 (8.5)	31.0 (19.6–48.9)	90 (37–220)	–	
Government employed	5 (1.0)	0.0	0.0	–	
Type of wall					
Stone/bricks/cement	143 (28.9)	25.9 (19.6–34.2)	96 (29–321)	–	
Clay/mud	195 (39.4)	29.7 (24.0–36.9)	85 (55–130)	–	
Wood	129 (26.1)	47.3 (39.4–56.8)	184 (113–299)	–	
Iron sheets	28 (5.7)	35.7 (21.5–59.2)	394 (114–1358)	–	
Source of water					
Piped/tap water	54 (10.9)	7.4 (2.9–19.2)	5 (1–18)	0.43 (0.10–1.83)	0.260
Borehole/well	71 (14.3)	52.1 (41.6–65.2)	369 (164–834)	0.74 (0.28–2.00)	0.554
Rainwater	6 (1.2)	66.7 (35.9–85.0)	792 (398–1576)	2.94 (0.42–20.58)	0.278
Stream/river	35 (7.1)	34.3 (21.5–54.6)	88 (33–233)	Reference	
Canal	327 (66.1)	33.0 (28.3–38.5)	94 (63–139)	1.10 (0.48–2.53)	0.826
Others	2 (0.4)	50.0 (7.0–55.0)	36 (5–256)	3.16 (0.15–65.63)	0.457

**Fig. 1:**
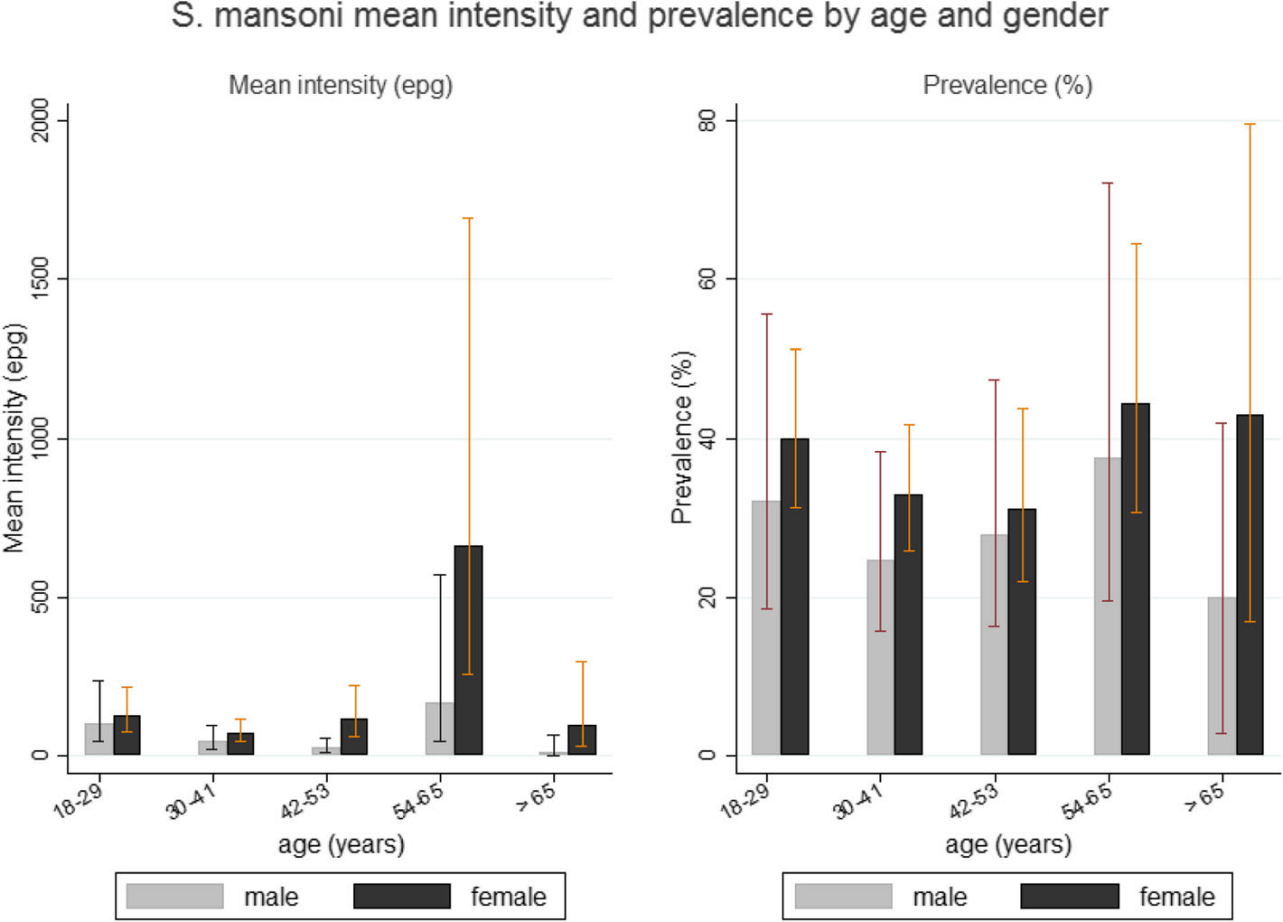
*S. mansoni* mean intensity and prevalence by age and gender

**Fig. 2:**
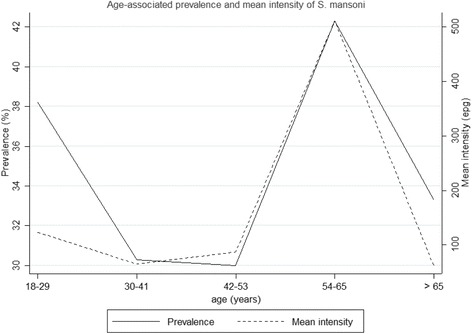
Age-associated prevalence and mean intensity of *S. mansoni*

Moreover, the prevalence and mean intensity of *S. mansoni* infection by age and gender are summarized in Fig. 1 showing that for all the age groups, females had higher prevalence of between 31.1 and 44.4%, compared to males who had a prevalence between 20 and 37.5%. The age-associated prevalence and mean intensity are also shown in Fig. 2, which showed that the highest levels of both prevalence and mean intensity of *S. mansoni* infections were among the participants aged 54–65 years.

The prevalence and mean intensity of STHs were almost zero, with hookworm being the only species present with a prevalence of 0.2% (95% CI 0–1.4) and mean intensity of epg (95% CI 0–0.1). Table 2 summarizes the prevalence of light, moderate and heavy intensity of *S. mansoni* infections. Overall, 6.9% (95% CI 5.0–9.5), 10.7% (95% CI 8.3–13.8) and 16.0% (95% CI 13.0–19.5) of the participants had heavy, moderate and light intensity of infections, respectively. A high number of the heavy infection cases were among the participants aged 54–65 years, 13.5% (95% CI 6.7–27.0), followed by those aged 18–29 years, 9.8% (95% CI 5.7–16.7).

**Table 2:** Prevalence (%) of light, moderate and heavy intensity of *S. mansoni* infection

Category	Classification of intensity of *S. mansoni* infection		
	Heavy	Moderate	Light
Overall	6.9 (5.0–9.5)	10.7 (8.3–13.8)	16.0 (13.0–19.5)
Gender			
Male	4.8 (2.3–9.9)	8.2 (4.8–14.2)	15.1 (10.2–22.2)
Female	7.7 (5.4–11.1)	11.7 (8.8–15.7)	16.3 (12.8–20.7)
Age group			
18–29	9.8 (5.7–16.7)	10.6 (6.3–17.7)	17.9 (12.2–26.2)
30–41	4.5 (2.4–8.6)	9.1 (5.8–14.1)	16.7 (12.2–22.8)
42–53	5.5 (2.5–11.9)	9.1 (5.0–16.5)	15.5 (10.0–24.0)
54–65	13.5 (6.7–27.0)	17.3 (9.5–31.5)	11.5 (5.4–24.7)
>65	0.0	25.0 (9.0–69.6)	8.3 (1.2–59.2)

Assessment of the factors associated with the helminth infections was determined for *S. mansoni* infection only, since the infection levels of STH infections were very low. After univariable analysis, all factors with *p* value of less than 0.3 were included in multivariable analysis with results as shown in Table 1. From this analysis, it was noted that village and the level of education of the participants were the only significant factors associated with *S. mansoni* infection. Study participants who reside in both Mianya A and B villages had significantly higher risk of infection with *S. mansoni*, aOR >2, *p* < 0.001. Similarly, those study participants with no education at all were twice as likely to be infected with *S. mansoni* compared to those who completed primary education, OR = 2.31, *p* = 0.039. However, the risk of *S. mansoni* infection reduced with the advancement in the level of education of the participant.

## Discussion

Few studies have been conducted to quantify the extent of schistosome and STH infections among adults living in areas of high endemicity. Results of the current study showed that there was high *S. mansoni* infections 33.5% (95% CI 29.6–38.0) and low STH infection 0.2% (95% CI 0–1.4) in adults living in Mwea West Sub-County. Current WHO guidelines recommend preventive chemotherapy based on regular anthelminthic drugs as a public health intervention to control helminthic infections and reduce morbidity [32]. Most of the public health programmes targeting STH and schistosome infections currently employ school-based deworming which has been previously shown to provide much benefits to untreated groups within and close to the treatment schools [33]. The findings of this study indicate that the adult community members of this area are also infected with these intestinal parasites, especially *S. mansoni* and may become a source of continued infection to school-age children, hence hindering the control programmes. Indeed, a study conducted in coastal region of Kenya showed that most control programmes for schistosome and STH infections often target school-age children only, leaving out the rest of the community members who are equally infected and may act as a reservoir for transmission and a source of re-infections to the school-age children [34]. The WHO Expert Committee on the Control of schistosome and STH infections during its meeting in 2001 noted that there is reduction in the cost of anthelminthic drugs generally and suggested that new options for treatment strategies should be considered [35]. Since praziquantel is now available at low cost (approximately US$0.2 per treatment), use of the drug more frequently may become possible [36]. The results of this study suggest that it may be necessary to develop other methods which can give preventive chemotherapy to adults in high endemic areas in order to reduce the disease transmission and enhance the control of the parasitic diseases being done in school-based deworming programmes. In most settings, it is estimated that 26–68% of individuals with schistosome infection are carriers of an additional helminthic infection, such as hookworms, *A. lumbricoides or T. trichiura* [37]. However, our study results showed that STH prevalence and intensity was quite low with 0.2% (95% CI 0–1.4). This can be attributed to regular treatment with albendazole by the Kirinyaga County, MOH, through the Community Health Extension Workers (CHEWs) who usually distribute the drugs quarterly in the study area.

In the current study, our results showed that *S. mansoni* infection was more predominant among those aged between 54 and 65 years, 13.5% (95% CI 6.7–27.0). This can mostly be attributed to the level of exposure noting that *S. mansoni* infections present as chronic disease in most cases, meaning that these age category might have high prevalence due to prolonged exposure to infection without treatment over the years. Another reason could be that most of these age category actively participate in rice farming in the rice paddies as opposed to other age cohorts.

In the present study, there was a significant association between education level and *S. mansoni* infection, aOR = 2.31 (95% CI 1.04–5.11), *p* = 0.039. This result concurs with a similar study conducted in western Côte D’Ivoire which found out that low education attainment was a risk factor associated with *S. mansoni* infection and low socioeconomic status for hookworm infection [38]. This could be due to improved knowledge in personal hygiene and sanitation, whereby those study subjects who are literate could be more knowledgeable on the pre-disposing factors that cause *S. mansoni* infection and are able to prevent/protect themselves through sanitation, personal hygiene and seeking health care. Other attributing factors could be environmental living circumstances whereby, in this area, majority of the illiterate and semi illiterate participate in rice farming which expose them to infection with *S. mansoni* infection. It is also widely acknowledged that people using natural freshwater and those pursuing specific occupations that expose them to open freshwater bodies such as rice farmers are at an elevated risk of schistosomiasis infection [39, 40]. In this study, a village (residential location) was also a risk factor closely associated with *S. mansoni* infection, whereby Mianya A and Mianya B showed significant association, aOR = 2.09 (95% CI 1.15–3.77), *p* = 0.015, and aOR = 4.38 (95% CI 2.02–9.48), *p* < 0.001, respectively. These results are in agreement with previous research findings, which clearly indicated that environmental living circumstances were closely linked to infection status and disease burden [41]. Consequently, additional ecological and environmental surveys are needed to understand the distribution and population patterns of the intermediate host (snail) which is directly related with the transmission of schistosomes. Indeed, environmental exposure due to location of residents rather than some established characteristics of an individual determines the risk of infection. This could also be due to the fact that the two villages are located within the irrigation scheme whereby the level below the ground is completely saturated with water (high water table) and, in most cases, there are no pit latrines within the rice paddies. This might have a major effect on latrine ownership, coverage and utilization and hence open defecation. Acka and colleagues reported a similar practice of poor hygiene, where villagers tended to defecate where convenient still rarely using latrines where available [42]. This practice allows helminth eggs from the feces of infected persons to contaminate the environment, including water sources and hence the community members. In this regard, it would be interesting to try a community-led total sanitation approach in the study area. This method can facilitate participation of community members in the study area to improve sanitation, hygiene practices, waste disposal and protection of drinking water sources [43].

The major potential weakness of this study may be the fact that only one stool sample was collected. The accuracy of the Kato-Katz technique in identifying individuals with *S. mansoni* infections and STHs is limited by day-to-day variation in egg excretion, and sensitivity is greatly reduced when intensity of infections is low [44]. This potentially leads to lower than actually detected infection prevalence and mean infection intensities. Improved detection of *S. mansoni* and STH eggs in stool requires examination of stool specimens collected on 2 to 3 consecutive days, which may not be practical especially when working in very remote areas. A new technique known as FLOTAC has been proposed as a better tool for diagnosis of parasitic infections like *S. mansoni*. In a study conducted in Cote d’Ivoire, the FLOTAC technique was found to have a sensitivity of 88.2% compared with 68.4% for Kato-Katz [45]. Another limitation of this study was that data on socio-economic status of the study participants was not collected, as this could have been used to rate the poverty levels in the study area. Finally, because the data results were only collected from villages within the irrigation scheme, we cannot make generalizations across the entire county of Kirinyaga and the small sample makes associations with helminth infection difficult to assess.

## Conclusions

The results of this study show that adult communities in Mwea West Sub-County were highly infected with *S. mansoni* and low infections with STHs. The study suggests that it may be necessary to include the adult population living in endemic areas, in MDA which usually targets primary schools for effective control programmes and reduce re-infections among the school children. We recommend that improved health education and awareness relating to hygiene practices, better sanitation and a more regular supply of piped water may help further decrease these parasitic infections and lead to better health status among the community members, hence an improved quality of life overall in the area and other endemic parts of the country.

## Additional file

Additional file 1: Table S1. Intensity thresholds for light, moderate and heavy infections with *Ascaries lumbricoides*, *Trichuris trichiura*, hookworms and schistosomes. (DOC 29 kb)

## Abbreviations

CHEWs: Community health extension workers
CI: Confidence interval
DALYs: Disability-adjusted life years
EPG: Eggs per gram
ESACIPAC: Eastern and Southern Africa Centre of International Parasite Control
GLS: Generalized least squares
KEMRI: Kenya Medical Research Institute
MDA: Mass drug administration
MOH: Ministry of Health
NGOs: Non-governmental organizations
NTDs: Neglected tropical diseases
OR: Odds ratio
PPS: Probability proportional to size
*S. mansoni*: *Schistosoma mansoni*
SERU: Scientific and Ethics Review Unit
STHs: Soil-transmitted helminths
WHO: World Health Organization
