# Supplementary material for: High prevalence of helminths infection and associated risk factors among adults living in a rural setting, central Kenya: a cross-sectional study
Source: Trop Med Health. 2017 Jul 1;45:15. doi: 10.1186/s41182-017-0055-8 (PMC5493853; doi:10.1186/s41182-017-0055-8)
Supplement: Additional file 1: Table S1. — Intensity thresholds for light, moderate and heavy infections with Ascaries lumbricoides, Trichuris trichiura, hookworms and schistosomes. (DOC 29 kb) [file 41182_2017_55_MOESM1_ESM.doc]

Table S1: Intensity thresholds for light, moderate and heavy infections with *Ascarieslumbricoides*, *Trichuristrichiura*, hookworms and schistosomes

| **Helminth** | **Intensity Threshold** | | |
| --- | --- | --- | --- |
|  | **Light** | **Moderate** | **Heavy** |
| *A. lumbricoides* | 1 – 4999 epg | 5000 – 49999 epg | ≥ 50000 epg |
| *T. trichiura* | 1 – 999 epg | 1000 – 9999 epg | ≥ 10000 epg |
| Hookworms | 1 – 1999 epg | 2000 – 3999 epg | ≥ 4000 epg |
| *S. mansoni* | 1 – 99 epg | 100 – 399 epg | ≥ 400 epg |
| *S. haematobium* | 1 – 50 eggs/10ml urine |  | ≥ 50 eggs/10ml urine |
